# Treatment patterns and outcomes in pancreatic cancer: Retrospective claims analysis

**DOI:** 10.1002/cam4.3011

**Published:** 2020-03-25

**Authors:** Yunes Doleh, Lincy S. Lal, Cori Blauer‐Petersen, Giovanni Antico, Michael Pishvaian

**Affiliations:** ^1^ AstraZeneca US Gaithersburg MD USA; ^2^ Optum Eden Prairie MN USA; ^3^ NCR Kimmel Cancer Center Sibley Memorial Hospital and Johns Hopkins University School of Medicine Washington DC USA; ^4^ Present address: Currently at Regeneron Pharmaceuticals Tarrytown NY USA

**Keywords:** cancer survival, healthcare costs, healthcare utilization, pancreatic cancer, retrospective study

## Abstract

**Background:**

Pancreatic cancer represents the third leading cause of US cancer deaths, with median survival <1 year. The goal of this study was to describe systemic treatments, healthcare utilization and costs, and overall survival among patients with unresectable/metastatic disease.

**Methods:**

This study used healthcare claims for commercial and Medicare Advantage enrollees diagnosed with pancreatic adenocarcinoma (at index date) during January 01 2010 to 31 May 2017. Included patients were aged ≥18 years, with continuous 6‐month preindex enrollment. Patients were excluded by resectable disease, another primary cancer, or pregnancy. Cohorts were based on first‐line (LOT1) chemotherapy regimen.

**Results:**

Overall, 12 978 patients (mean age 70 years, 51% male) were included, among which 5610 (43%) received chemotherapy. Of those, 23% received gemcitabine monotherapy, 22% gemcitabine‐*nab* paclitaxel, 22% FOLFIRINOX, 3% FOLFOX, and 29% received other regimens. Mean LOT1 duration was 112 days; 60% did not undergo subsequent lines of therapy. Moreover, 50% of patients had an emergency room visit and 45% were hospitalized during LOT1. Among treated and untreated patients, mean total 6‐month costs were $52 101. We found that patients receiving FOLFIRINOX had the highest costs, whereas those who received gemcitabine monotherapy had the lowest. Median overall survival (mOS) was 335 days with any first‐line treatment. FOLFIRINOX‐treated patients had the highest mOS (492 days), whereas gemcitabine monotherapy‐treated patients had the lowest (223 days).

**Conclusions:**

A large proportion (57%) of patients with unresectable/metastatic pancreatic cancer did not receive chemotherapy. Healthcare costs were higher for fluorouracil‐based regimens, while lower for gemcitabine‐based regimens. Survival rates were within expectations for advanced pancreatic cancer.

## Graphical abstract

In a real‐world retrospective study, 57% of patients with advanced metastatic pancreatic cancer did not receive chemotherapy. Total healthcare costs were higher for patients treated with fluorouracil‐based regimens, and lower with gemcitabine‐based regimens.

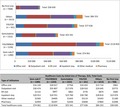

## 1 INTRODUCTION

In the United States (US), pancreatic cancer is currently the third leading cause of cancer deaths, and is estimated to become the second leading cause by 2030.^1^ For 2020, pancreatic cancer estimates include 57 600 new cases diagnosed and 47 050 deaths due to the disease.^2^ Although rates of pancreatic cancer diagnoses have increased slightly in recent years, survival statistics have not improved significantly.^3^ This is true despite recent developments in chemotherapeutic options, in part because most patients can have asymptomatic advanced or metastatic disease. Relative 5‐year survival of all types of pancreatic cancer was 9.3% between 2009 and 2015; for patients with advanced or metastatic disease, the rate is 2.9%.^3^

The majority of pancreatic tumors, more than 85%, are adenocarcinomas arising from the ductal epithelium.^4^ Fewer than 20% of cases of pancreatic cancers present with localized disease that is surgically resectable.^4^ For most patients with advanced unresectable or metastatic pancreatic cancer, clinical trial enrollment should be offered.^5^ Outside of a clinical trial, the initial preference is to start with chemotherapy, according to American Society of Clinical Oncology (ASCO) and European Society for Medical Oncology (ESMO) guidelines.^5^, ^6^ The optimum regimen is not established, but may include FOLFIRINOX (fluorouracil, leucovorin, irinotecan, and oxaliplatin), gemcitabine‐plus *nab*‐paclitaxel, or in patients with a poor performance status, gemcitabine monotherapy. Chemoradiotherapy can also be used for palliative purposes as initial therapy, with concomitant fluoropyrimidine‐based and gemcitabine‐based approaches.

Observational studies evaluating clinical and economic outcomes in advanced (nonresectable) pancreatic cancer are limited. DaCosta Byfield and colleagues estimated costs and resource utilization associated with pancreatic cancer in a commercially insured US sample.^7^ The mean total per‐patient‐per‐month (PPPM) costs were $15 480, and for the metastatic disease treatment phase, $21 637.

Significant gaps exist in the literature comparing currently available agents in locally advanced unresectable and metastatic pancreatic cancer treated outside of the clinical trial setting. This study was performed to understand current treatment patterns, healthcare resource use and costs, and survival outcomes in “real world” clinical practice. The specific objectives were to describe (a) newly diagnosed patients with locally advanced unresectable/metastatic pancreatic adenocarcinoma; (b) systemic regimen treatment patterns by cohorts identified by initial line of therapy (LOT1); (c) healthcare resource utilization and costs up to 6 months' follow‐up; and (d) overall survival.

## 2 METHODS

### 2.1 Study design

This was a retrospective database study examining treatment patterns and outcomes among pancreatic adenocarcinoma patients with locally advanced/metastatic disease. The study used claims‐based medical data, pharmacy data, enrollment information, and mortality data from 01 July 2009 to 31 May 2017. Study subjects were commercial and Medicare Advantage enrollees with a diagnosis of pancreatic adenocarcinoma during the identification period of 01 January 2010 to 31 May 2017. Additional outcomes measures included healthcare utilization, costs, and survival.

All patients were required to be continuously enrolled in the health plan for at least 6 months (preindex period) before the first claim with a diagnosis code for advanced disease. During this period, preindex characteristics were described. The variable period following the index date was used to assess outcomes including healthcare utilization and costs.

### 2.2 Data sources

#### 2.2.1 Medical and pharmacy claims data

Data regarding pharmacy and medical claims were accessed via a proprietary database, Optum Research Database, which contains medical and pharmacy claims data for patients insured by commercial and Medicare Advantage health plans. Medical claims include diagnosis and procedure codes, and paid amounts, collected from all sites of healthcare. Pharmacy claims are obtained from outpatient prescription fills.

#### 2.2.2 Mortality data

Mortality data were obtained from several sources: the Social Security Administration Death Master File (SSADMF); the National Death Index (NDI); and the Center for Medicare and Medicaid Services (CMS or MCR); and healthcare claims, as available. The NDI and CMS sources provide timing of deaths not always captured in claims and data that may be missing in the SSADMF. With proper linkage, CMS files establish date of death with a 1‐month lag time, but not cause of death. The CMS death information was sourced from the CMS Health Insurance Claim Number to Medicare Beneficiary Identifier crosswalk. The NDI, a central index of death record information from state vital statistics offices, including cause of death. The NDI early release version has a lag time of approximately 6 months. Approval through the Optum data disclosure analysis process was required for the use of exact death dates. Only fully insured and nongroup Medicare Advantage enrollees were matched to the NDI for this study: 65% of the study sample who met all inclusion and exclusion criteria were matched per compliance rules; death data were searched for up to a 2‐year period after the last active claim date.

#### 2.2.3 Patient privacy and protocol review

Data were de‐identified in compliance with the HIPAA Privacy Rule when disclosed for this research. Appropriate research and ethical reviews took place prior to linking with NDI database. Following linkage for mortality, data were de‐identified prior to delivery to the research team for analysis. No patient's identity or medical records were disclosed for the purposes of this study except in compliance with applicable law.

### 2.3 Inclusion and exclusion criteria

To be included in the study, subjects were at least 18 years of age, and had at least two non‐diagnostic claims for pancreatic cancer in any claim position on two separate days during the study identification period (with date of first of these claims set as the index date), and had to have evidence of metastatic disease by the index date or within 60 days of index date. Patients were flagged for treatment with anticancer systemic therapy: at least 1 claim for a systemic/immuno‐oncologic therapy during the identification period, after the index date. These included both National Comprehensive Cancer Network‐approved and nonapproved pancreatic cancer drugs in the first line of therapy. See Appendix for diagnostic codes (Tables S1 and S2) and procedure (Table S3) codes for systemic therapy administration.

In addition, continuous enrollment with medical and pharmacy benefits for at least 6 months (180 days) prior to the index date (baseline period) and including the index date was required. Variable follow‐up ended on the earliest date of disenrollment from the health plan or 31 May 2017 or death. The start date of continuous enrollment before the index date (as early as 01 July 2009) was evaluated to verify a 6‐month period free of pancreatic cancer.

Patients were excluded by the following criteria: at least 1 claim for pancreatic cancer‐specific surgery (eg, pancreaticoduodenectomy) before the first line of treatment; 1 claim for clinical trial drug in the baseline period; or evidence of other primary cancers (based upon systemic treatment) during baseline. Patients with evidence of pregnancy during the baseline period were also excluded.

### 2.4 Measures and outcomes

#### 2.4.1 Patient characteristics

Baseline demographic characteristics were obtained, including the index month/year, patient age as of the index year, gender, insurance type (commercial or Medicare Advantage), and geographic region, in accordance with the US Census Bureau region designations.^8^ The mean (±standard deviation [SD]) Quan‐Charlson comorbidity score was calculated based on the presence of diagnosis codes on medical claims in the preindex period, including diagnosis of metastasis.^9^

#### 2.4.2 Exposures

The start of the first LOT was identified at the date of first received systemic anticancer therapy, including all chemotherapeutic agents filled/infused within the first 30 days of the start date. The end of the LOT was identified as the earliest of any of the following: (a) start of a new regimen as indicated by initiation of a new agent; (b) gap in therapy or discontinuation of all agents in the first regimen (eg, ≥60 days of no regimen agents); (c) end of the study period/disenrollment; or (d) death. Note that a LOT that ended because of disenrollment was considered incomplete.

The systemic treatment regimen in the first LOT determined the exposure cohorts. Patients were assigned to a study cohort based on the observed initial treatment regimen, primarily including gemcitabine plus nanoparticle albumen‐bound (*nab*)‐paclitaxel (Gem‐*nab*‐P); FOLFOXIRI/FOLFIRINOX (includes folinic acid [leucovorin], fluorouracil, irinotecan, and oxaliplatin); gemcitabine monotherapy; or folinic acid (leucovorin), fluorouracil, and oxaliplatin (FOLFOX). A category of “Others” was based upon any other regimen of drugs observed. In addition, outcomes were determined among patients who did not receive any systemic therapeutic regimen during the study period (“No Line 1 treatment”).

#### 2.4.3 Outcomes

##### Healthcare utilization and costs

All‐cause healthcare utilization (as PPPM counts) was calculated over a 6‐month period. Healthcare costs were measured as total amounts over 6 months, and as PPPM amounts for 6 months and the full follow‐up period. Utilization and costs are reported for patients with at least 6 months of follow‐up postindex (or less if the patient died within the first 6 months). Utilization and costs were calculated for ambulatory visits (office and outpatient), emergency room (ER) visits, and inpatient stays. Costs included health plan‐ and patient‐paid amounts, adjusted using the annual medical care component of the Consumer Price Index for inflation between 2010 and 2016.^10^ Coordination of benefits payments was included in the costs.

##### Survival

To determine overall survival, the number of days was summed from the index date to death date as evidenced by death per SSADMF, NDI, CMS, or claims. Patients were censored at the end of the study period or the disenrollment date. Death was counted as an event within the main analyses.

##### Survival sensitivity analysis

In a sensitivity analysis to determine the effect on overall survival of censoring, patients were considered censored if they were alive at the end of the study period or disenrollment date, or 1 year post the last claims date, and were not linkable to external death data sources (NDI, CMS). Noncensored patients included those who had died or were linked to NDI/CMS, with or without evidence of death.

### 2.5 Statistical analyses

#### 2.5.1 Descriptive analysis

All study variables, including preindex and outcome measures, were analyzed descriptively. Count and proportions (n, %) were provided for dichotomous and polychotomous variables. Mean (SD) and median were provided for continuous variables. Descriptive techniques that account for length of observation time (PPPM amounts) were used where appropriate. Differences across all cohorts were determined using Chi‐square test and analysis of variance (ANOVA).

#### 2.5.2 Kaplan‐Meier analysis

Kaplan‐Meier analysis was used to estimate overall survival. It was used to measure the fraction of patients surviving for a certain amount of time after treatment. Survival data were stratified based upon first‐line treatment regimens (or no treatment). Log‐rank test on the probability of death was performed to determine differences across the cohorts, with *P* < .05 indicating statistical significance.

## 3 RESULTS

### 3.1 Study sample characteristics

**FIGURE 1:**
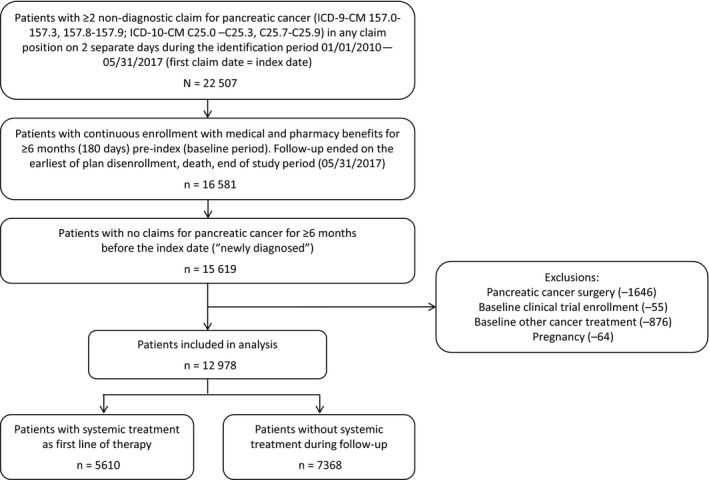
Patient identification and attrition flow chart. Patients were included by evidence of pancreatic cancer, continuous enrollment in their health plans, and a 6‐month baseline period with no claims for pancreatic cancer. Exclusion criteria were based upon evidence of pancreatic cancer surgery, clinical trial enrollment, or evidence of pregnancy. ICD‐9‐CM/‐10‐CM, *International Classification of Diseases, Clinical Modification, 9th and 10th Revisions*

Among the final analytic sample of 12 978 patients, 5610 (43.3%) had evidence of systemic treatment for pancreatic cancer and 7368 (56.8%) patients did not receive any systemic anticancer treatment (chemotherapy) in the follow‐up period (Figure 1). Among patients included in the analyses (n = 12 978), 1322 (10.2%) had gemcitabine monotherapy; 1280 (9.9%) had gemcitabine plus *nab*‐paclitaxel (Gem‐*nab*‐P); 1234 (9.5%) had FOLFIRINOX; 161 (1.2%) had FOLFOX; and 1613 (12.4%) had other systemic treatment, with the highest percentage having capecitabine (9.7%) or gemcitabine‐cisplatin (9.5%) (Table S4). Among patients in the “other” group, 25% received a combination which included a platinum‐based agent.

Selected demographic and clinical characteristics by first line of therapy are shown in Table 1. The total sample of patients had mean age of approximately 70 years with a nearly equal female‐to‐male ratio (49:51, respectively), a majority (65%) had Medicare Advantage insurance coverage, and 71% were located in the Midwest and South regions of the United States. Among all patients, the mean (SD) comorbidity score was 2.86 (2.94) with a median score of 2.00. Among all patients included in the study, 96.7% had distant metastasis and 2.3% had lymph node metastasis only at baseline. Per study design, no patients had surgical resection. The median continuous follow‐up was 188 days, among the entire sample, with a median of 116 days for the no treatment group.

**TABLE 1:** Characteristics of the Analytic Sample, by LOT1 Regimen

	Total (N = 12 978)	Gem‐*nab*‐P (n = 1280)	FOLFIRINOX (n = 1234)	Gemcitabine (n = 1322)	FOLFOX (n = 161)	Others (n = 1613)	No first line (n = 7368)	*P*‐value*
Age (continuous) mean (SD)	70.3 (11.50)	69.7 (9.0)	62.5 (8.9)	72.0 (9.5)	65.8 (9.8)	66.0 (11.1)	72.5 (11.9)	<.001
Median	71.0	71.0	63.0	73.0	66.0	67.0	75.0	
Interquartile range	17.0	12.0	12.0	13.0	12.0	15.0	16.0	
Gender, female, %	49.4	48.2	41.5	49.9	38.8	46.8	51.6	<.001
Coverage type, n (%)								<.001
Commercial	4524 (34.9)	412 (32.2)	770 (62.4)	416 (31.5)	77 (47.8)	800 (49.6)	2049 (27.8)	
Medicare advantage	8450 (65.1)	867 (67.7)	463 (37.5)	906 (68.5)	84 (52.2)	811 (50.3)	5319 (72.2)	
Geographic region, n(%)								<.001
Northeast	2272 (17.5)	190 (14.8)	192 (15.6)	208 (15.7)	36 (22.4)	248 (15.4)	1398 (19.0)	
Midwest	3938 (30.3)	441 (34.5)	475 (38.5)	497 (37.6)	47 (29.2)	418 (25.9)	2060 (28.0)	
South	5301 (40.9)	491 (38.4)	445 (36.1)	470 (35.6)	61 (37.9)	760 (47.1)	3074 (41.7)	
West	1461 (11.3)	156 (12.2)	122 (9.9)	146 (11.0)	16 (9.9)	187 (11.6)	834 (11.3)	
Baseline Charlson comorbidity score^1^								<.001
Mean (SD)	2.86 (2.94)	2.93 (3.00)	2.38 (2.70)	2.97 (3.11)	3.55 (3.19)	3.31 (3.08)	2.80 (2.88)	
Median	2.00	2.00	2.00	2.00	2.00	2.00	2.00	
Metastasis type, n(%)								.485
Any distant metastasis	12 552 (96.7)	1239 (96.8)	1190 (96.4)	1289 (97.5)	154 (95.7)	1553 (96.3)	7127 (96.7)	
Lymph only	426 (3.3)	41 (3.2)	44 (3.6)	33 (2.5)	7 (4.3)	60 (3.7)	241 (3.3)	
Available follow‐up								<.001
Continuous (days) mean (SD)	423 (560)	318 (296)	416 (378)	357 (470)	392 (465)	492 (534)	440 (635)	
Median	188	231	311	202	234	296	116	

Abbreviations: FOLFIRINOX, folinic acid [leucovorin], fluorouracil, irinotecan, and oxaliplatin; FOLFOX, folinic acid (leucovorin), fluorouracil, oxaliplatin; Gem‐*nab*‐P, gemcitabine plus *nab*‐paclitaxel; LOT1, first line of treatment; SD, standard deviation.

* *P* < .05 indicates significant differences across six subgroups of patients treated and untreated.

### 3.2 Treatment patterns

**FIGURE 2:**
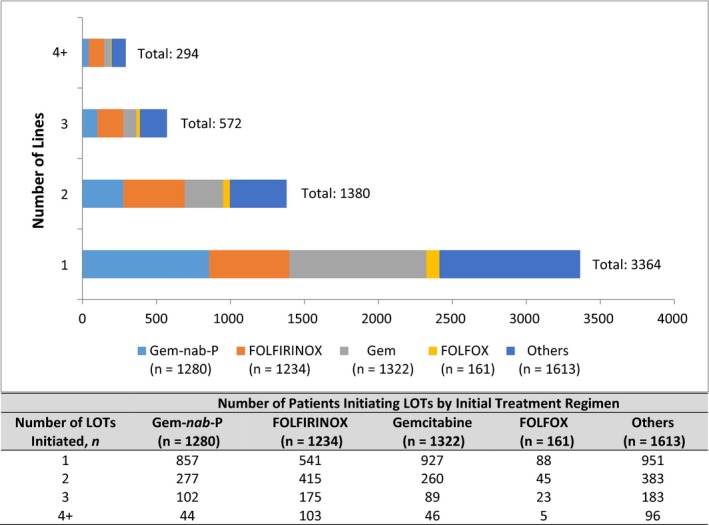
Numbers of patients who had 1, 2, 3, or 4+ total regimens, as identified by their first line of systemic treatment regimen (LOT1). Among the patients who initiated at least one, 60% had only one LOT, 25% had two, 10% had three, and 5% had four LOTs during the study period. FOLFIRINOX, folinic acid [leucovorin], fluorouracil, irinotecan, and oxaliplatin; FOLFOX, folinic acid (leucovorin), fluorouracil, oxaliplatin; Gem‐*nab*‐P, gemcitabine plus nab‐paclitaxel; gemcitabine alone; or others

Gemcitabine (23.6%), Gem‐*nab*‐P (22.8%), and FOLFIRINOX (22.0%) were the most commonly selected regimens for LOT1; median treatment durations (including censored lines) by LOT1 regimen are shown in Table 2. Those treated with Gem‐*nab*‐P had the longest median treatment duration (93 days); excluding censored lines raised the median to 99 days. Median durations were 85 days for FOLFIRINOX, 71 days for gemcitabine monotherapy, and 65 days for FOLFOX. Among patients receiving other treatments, the median duration of therapy was 76 days. Among patients who initiated treatment with a systemic LOT (n = 5610), Figure 2 shows the numbers of patients who had 1, 2, 3, or 4+ total regimens, as identified by their initial LOT. Among the patients who initiated one systemic LOT, 60% had only one LOT, 25% had two, 10% had three, and 5% had four LOTs during the study period.

**TABLE 2:** Treatment Durations by LOT1 Regimen

		Total (N = 5610)	Gem‐*nab*‐P (n = 1280)	FOLFIRINOX (n = 1234)	Gemcitabine (n = 1322)	FOLFOX (n = 161)	Others (n = 1613)
Treatment duration (d) including censored lines	n	5610	1280	1234	1322	161	1613
	Mean (SD)	112 (111)	124 (106)	119 (118)	97 (95)	88 (71)	111 (122)
	Median	81	93	85	71	65	76
Treatment duration (d) excluding censored lines	n	3951	787	911	952	121	1180
	Mean (SD)	113 (101)	133 (110)	122 (104)	97 (87)	87 (65)	107 (104)
	Median	84	99	91	70	65	76

Abbreviations: FOLFIRINOX, folinic acid [leucovorin], fluorouracil, irinotecan, and oxaliplatin; FOLFOX, folinic acid (leucovorin), fluorouracil, oxaliplatin; Gem‐*nab*‐P, gemcitabine plus *nab*‐paclitaxel; LOT, line of treatment; SD, standard deviation.

### 3.3 Healthcare resource utilization and costs

**FIGURE 3:**
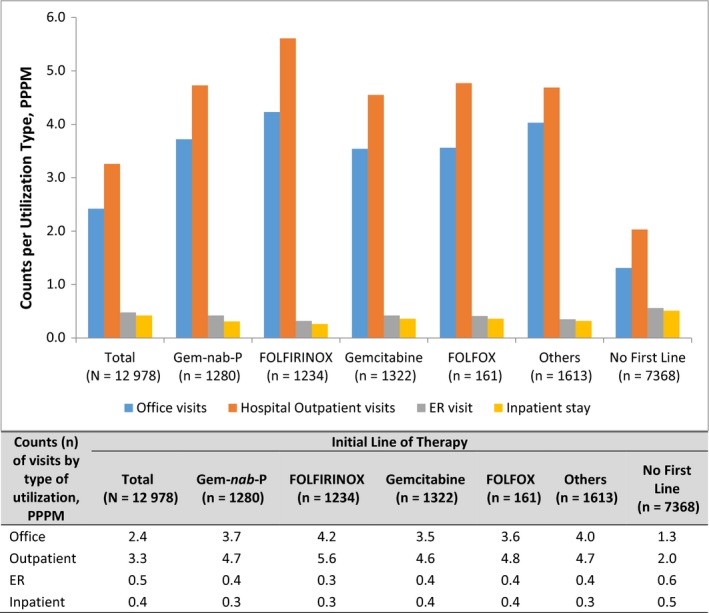
Healthcare resource utilization for up to 6 mo postdiagnosis, by first line of systemic treatment (LOT1) regimen. Per‐patient‐per‐month (PPPM) counts of utilization types by regimen: Gem‐*nab*‐P, gemcitabine plus *nab*‐paclitaxel; FOLFIRINOX, folinic acid [leucovorin], fluorouracil, irinotecan, and oxaliplatin; FOLFOX, folinic acid (leucovorin), fluorouracil, oxaliplatin; gemcitabine alone; or others, or no first‐line systemic treatment (Tx); ER, emergency room

**FIGURE 4:**
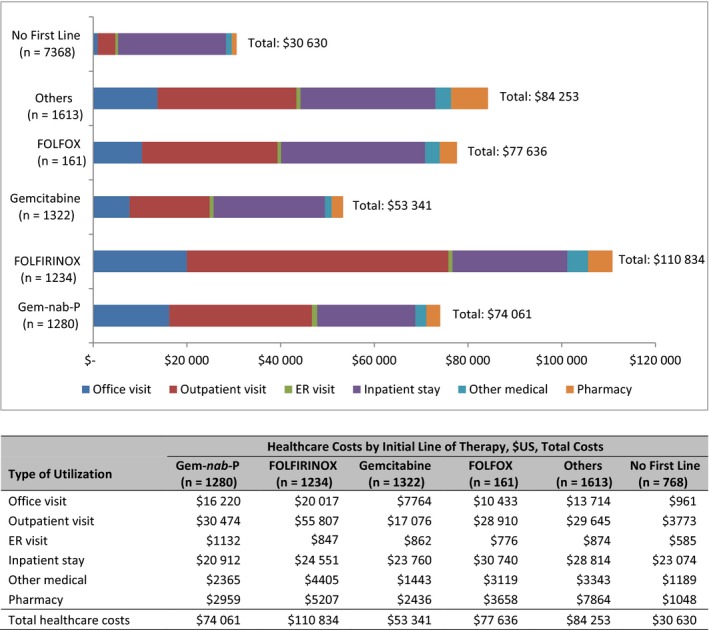
All‐cause mean monthly healthcare costs (by type: office visits, outpatient visits, ER visits, inpatient stays, other medical costs, pharmacy costs, and total) for up to 6 mo postdiagnosis by first line of systemic treatment (LOT1) regimen. ER, emergency room; FOLFIRINOX, folinic acid [leucovorin], fluorouracil, irinotecan, and oxaliplatin; FOLFOX, folinic acid (leucovorin), fluorouracil, oxaliplatin; Gem‐*nab*‐P, gemcitabine plus *nab*‐paclitaxel; gemcitabine alone; or others, and no first‐line treatment (Tx); US, United States

The use of healthcare resources (Figure 3) and their associated costs (Figure 4) were determined for patients receiving a first LOT and those not receiving any systemic treatment for the 6‐month (or less for patients who died) postdiagnosis period. Those in the FOLFIRINOX group had the highest counts of hospital outpatient and office visits. Patients with no treatment had the highest counts of ER and inpatient visits. The mean total all‐cause costs among all patients (treated and untreated) were $52 101 over 6 months postdiagnosis. Those in the FOLFIRINOX cohort had the highest costs ($110 834), with the greatest proportion (50%) attributable to outpatient care. Patients in the gemcitabine‐only cohort had the lowest ($53 341), with 32% attributable to outpatient care and 45% attributable to inpatient care. Among all patients studied, those with no first line of systemic therapy had the lowest costs ($30 630), with the greatest proportion attributable to inpatient care. The PPPM values for the 6‐month period and the full follow‐up period were similar (Table S5).

### 3.4 Overall survival

**FIGURE 5:**
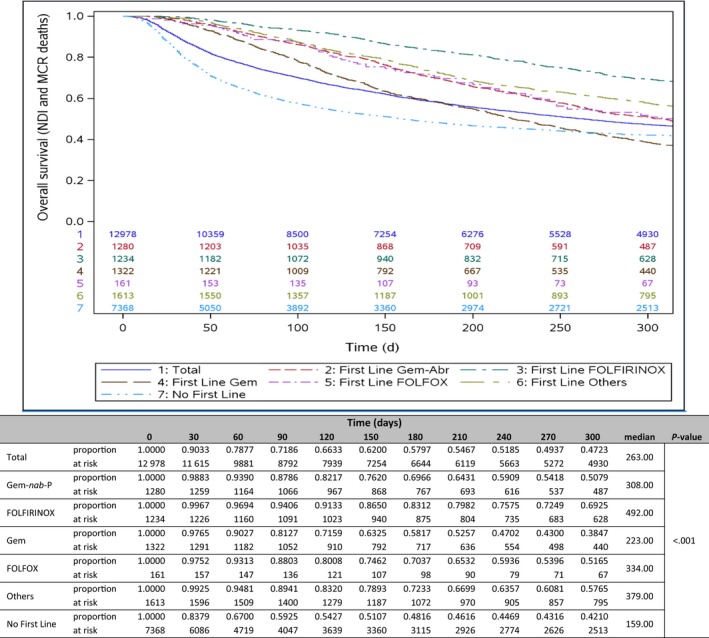
Overall survival analysis: risk at intervals by first‐line regimen. Kaplan‐Meier curve comparing overall survival across first line of systemic treatment (LOT1) regimens: Gem‐*nab*‐P, gemcitabine plus *nab*‐paclitaxel; Gem, gemcitabine; FOLFIRINOX, folinic acid [leucovorin], fluorouracil, irinotecan, and oxaliplatin; FOLFOX = folinic acid (leucovorin), fluorouracil, oxaliplatin; others; and no first‐line treatment (Tx). NDI, National Death Index; MCR or CMS, Center for Medicare and Medicaid Services

**FIGURE 6:**
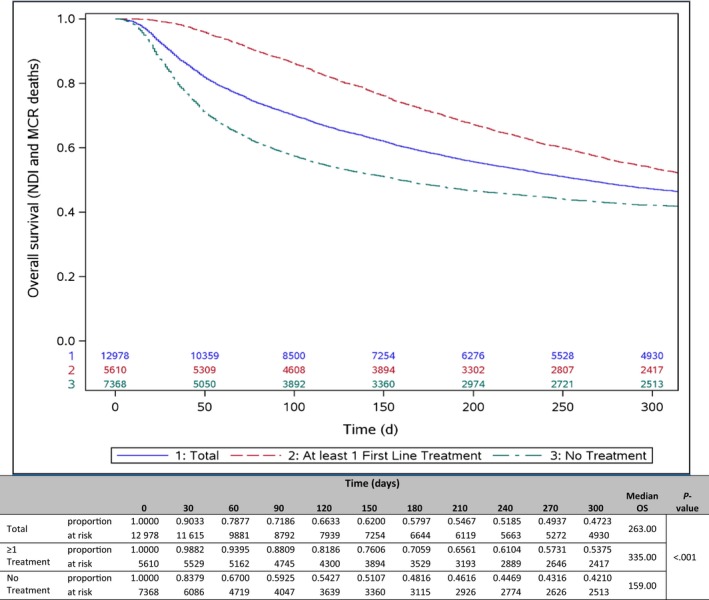
Overall survival analysis for any first‐line treatment versus no first‐line treatment. Kaplan‐Meier curve comparing total patients, patients with at least one line of treatment (LOT), and patients with no systemic treatment (Tx). MCR or CMS, Center for Medicare and Medicaid Services; NDI, National Death Index

Among all patients, the median overall survival (mOS) was 263 days (8.8 months). The FOLFIRINOX cohort had a mOS of 492 days (16.4 months), those receiving “Other” regimens had 379 days (12.6 months), and the Gem‐*nab*‐P cohort had 308 days (10.3 months). In contrast, patients receiving gemcitabine only had a median 223 days (7.4 months) overall survival, and patients who received no first‐line regimen had a median of 159 days (5.3 months). Figure 5 provides the Kaplan‐Meier proportions for overall survival by first‐line treatment regimen. The proportion at risk at 300 days was 0.47 among all patients, with significant differences detected across all cohorts (*P* < .001). The survival curves (Figure 6) are significantly different (*P* < .001) between patients who received any treatment (median 335 days [11 months]) and those who received no treatment (159 days [5.3 months]).

### 3.5 Sensitivity analysis: survival

**FIGURE 7:**
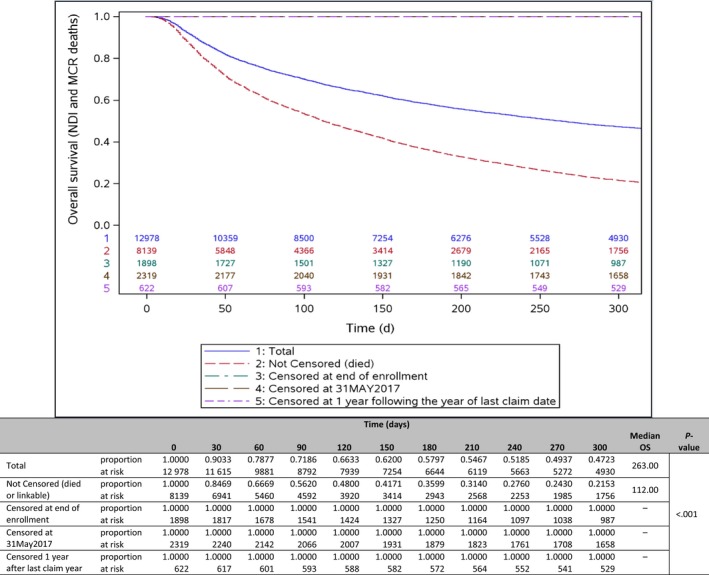
Overall survival sensitivity analysis. Kaplan‐Meier curve comparing total patients, patients with evidence of death, and patients censored by end of continuous health plan enrollment, end of study date, or at 1 y following the year of the last claim date. All censored groups are included in the line at the top of the chart. MCR or CMS, Center for Medicare and Medicaid Services; NDI, National Death Index

The sensitivity analysis examined percent of patients who were not censored, that is, patients who died (during the study period) or were available for linkage with CMS or NDI database versus patients who were censored (no evidence of death) by end of enrollment, study end date, or 1‐year after last claim date (Figure 7). Among all patients, 63% were not censored and 37% were censored during the study period. While 47% of the total population was still alive for the total study cohort, in the population that were not censored, only 22% was alive after including all the external sources of death data.

## 4 DISCUSSION

Despite recent developments in chemotherapeutic treatment of pancreatic cancer, the prognosis remains poor overall, especially for advanced unresectable/metastatic disease. Optimizing treatment toward the most cost‐effective regimen for survival benefit is a high priority in pancreatic cancer care. In order to evaluate options, data obtained both in clinical trial and routine practice settings are needed to analyze treatment course, healthcare utilization and costs, and survival. This study evaluated these measures among a sample of patients enrolled in commercial and Medicare Advantage health plans, who were treated outside of clinical trials, for unresectable/metastatic disease. It should be noted that, in our analysis, the vast majority of patients had evidence of metastatic disease. These results can be utilized to compare to clinical trials and other reports of patients with metastatic disease.

### 4.1 Treatment patterns

Overall, 12 978 patients met criteria for the study, and 5610 patients had a first‐line systemic treatment, the most common being gemcitabine monotherapy, gem‐*nab*‐P, FOLFIRINOX, and FOLFOX; and 29% of treated patients had others (including the highest percentage having capecitabine and next highest gemcitabine plus cisplatin). Mean treatment duration was 112 days, but 60% did not start a second line. The sample varied somewhat in age and insurance coverage type by treatment option, with patients receiving FOLFIRINOX being the youngest patients with the highest percentage enrolled in commercial plans, as well as having the lowest baseline Quan‐Charlson comorbidity score. Their geographic distributions were consistent with the overall database and with the US Medicare Advantage and commercially insured population.^11^

Of particular note, 57% of patients did not receive a first‐line chemotherapeutic regimen. However, the median follow‐up time for untreated patients was 116 days, far shorter than the follow‐up times (202‐311 days) available for patients who were treated. Although the mean age among these patients was higher than the other regimen groups, it remains unclear whether age, comorbid status, or any other variables may have influenced the patients' choices to pursue chemotherapy. Recent studies have explored reasons for patients with advanced pancreatic cancer not receiving treatment. For example, one study demonstrated regional variation in receipt of treatment at all stages; among patients with stage IV disease, more than 40% received no treatment.^12^ A SEER study among Medicare patients observed that patients with advanced cancer, older age, and affected by poverty were more likely to receive no treatment.^13^ However, in contrast to this study, their sample of patients comprised only 25% with distant metastases and the study included surgical‐ and radiation‐treated patients.

The realization that >50% of newly diagnosed advanced pancreatic cancer patients never received any systemic therapy in itself is alarming. There may be many reasons for this finding. One would be that the financial costs associated with the treatments, even in the insured population, are too high and the patients are choosing not to receive treatment due to inability to pay the copay or coinsurance associated with the treatment. It would also be concerning, but not inconceivable to think that these patients are not being treated due to the nihilism surrounding a pancreatic cancer diagnosis. Efforts to educate patients, as well as first medical points of contact (eg, primary care physicians, and gastroenterologists) on the proven benefits of systemic therapy must become a priority.

### 4.2 Healthcare utilization and costs

Mean total costs among all patients (treated and untreated) were $52 101 over 6 months postdiagnosis. Those in the FOLFIRINOX cohort had the highest costs, with approximately half attributable to outpatient care. Patients receiving gemcitabine‐based regimens had the lowest total costs, with approximately one third attributable to outpatient care. Among all patients studied, those with no first line of systemic therapy had the lowest total costs, but the largest proportion of costs attributable to inpatient care. The findings of this study are in alignment with expectations in terms of treated versus untreated patients, although comparison across retrospective studies is challenging due to variation in study period, insurance coverage of the study population, design, endpoints, and patient samples.

The only recent US real‐world study evaluating costs included metastatic cancer patients treated with first‐line Gem‐*nab*‐P or FOLFIRINOX.^14^ Supportive care costs and inpatient hospitalization rates were significantly lower with Gem‐*nab*‐P, although costs of drug acquisition were higher. Total costs of care PPPM, adjusted for covariates, were lower for patients treated with Gem‐*nab*‐P. In this study, no direct comparison was made between individual drugs, yet the magnitude of PPPM costs was similar to those in the comparison study.^14^ The FOLFIRINOX cohort in this study had the highest PPPM outpatient costs.

Although systemic cancer treatment in general is costly,^14^ the majority of costs among all patients in this study was for medical care, rather than pharmacy costs. In a 2013 study, total PPPM costs among a sample of patients with pancreatic cancer were comparable to those in this study, although their sample included all stages and did not distinguish specific systemic therapies.^7^ However, among patients in a metastatic phase, the largest proportion of mean costs were due to management of complications, rather than chemotherapy, similar to our study. Further study of costs associated with specific regimens is warranted, especially among older patients with advanced disease, for whom out‐of‐pocket costs, as well as toxicity, may be weighed against overall benefit.^15^

### 4.3 Survival

Among the entire sample of 12 978 patients included in this study, the mOS was 263 days (8.8 months); however, this figure includes 7368 patients who had no first‐line systemic therapy. For those patients, median survival was 159 days (5.3 months). The longest mOS was 492 days (16.4 months) in the FOLFIRINOX cohort in this study, and the lowest was observed among patients receiving gemcitabine only at 223 days (7.4 months). Data for patients included in this study were obtained over the years 2009‐2017. The most relevant clinical trials of systemic regimens across similar years reported survival data comparing fluorouracil‐based regimens and gemcitabine‐based regimens. For example, the PRODIGE study demonstrated higher overall survival with FOLFIRINOX compared with gemcitabine alone (11.1 months vs 6.8 months, respectively).^16^ The addition of *nab*‐paclitaxel to gemcitabine increased the median length of overall survival (8.5 months) compared with gemcitabine alone (6.7 months) as observed in the MPACT trial.^17^ Similarly, in this study, the Gem‐*nab*‐P cohort also had a longer median survival of 308 days (10.3 months) than patients with gemcitabine only (223 days; 7.4 months).

Comparing survival data from clinical trials to those of the current retrospective study is hindered by differences in study design, sample characteristics, and available data on deaths. However, similar limitations hinder comparisons among regimens with recent retrospective studies and systematic reviews/meta‐analyses.^18^, ^19^, ^20^, ^21^, ^22^ Thus, conclusions regarding superiority of any one regimen are problematic. In a 2018 meta‐analysis, Hall and colleagues report that although survival rates have improved minimally over the past 30 years, only FOLFIRINOX has a weighted mOS over 10 months in clinical trials.^23^

Of particular note, patients in this study who received no first‐line regimen had a mOS of 159 days (5.3 months). This difference represents a substantial gap in the use of systemic treatment among patients with metastatic pancreatic cancer.

### 4.4 Limitations

Limitations of this study included those inherent with the use of administrative claims, including possible coding errors and unrecorded care received outside the health insurance plan. Potential confounders, including performance status, stage, histology, and molecular biomarker status, were not available for analysis and would have impacted outcomes. Furthermore, provider and formulary characteristics, as well as information about patients' wishes or providers' approach, that may have influenced medication access and choice, were not available. Additionally, costs used in this analysis as paid costs to the providers, that is, the reimbursed amount based on negotiated rates; and serve only as a proxy for the real costs of medical care in this country.

Traditionally, death date may be missing for up to 50% of the deaths, as the SSA DMF no longer provides death data sourced from “electronic data capture” for secondary research purposes. This limitation was addressed by matching with the NDI database and addition of the CMS data source. In the population that had evidence of death or were available for linkage to all the sources of data, the percentage that survived is in line with literature and the median survival times are also in line with current clinical trial literature. Finally, these results were from a managed care population in the US and may not be generalizable to other populations, such as uninsured and those with traditional Medicare insurance.

## 5 CONCLUSIONS

In this study, treatment patterns and overall survival rates were in line with expectations for advanced pancreatic cancer. Although direct comparisons were not performed between individual regimens, patients receiving gemcitabine monotherapy had the lowest total costs and those receiving FOLFIRINOX had the highest costs, among all patients who received first‐line treatment. Notably, 57% of patients in this study did not receive first‐line systemic therapy. Among patients who did receive first‐line therapy, 60% did not receive subsequent therapy. These findings indicate a significant gap exists for future treatment options to fill.

## CONFLICT OF INTEREST

CB‐P and LL report employment with Optum, but no other conflicts of interest. YD reports employment and stock ownership with AstraZeneca, and stock ownership with Bristol‐Myers Squibb. GA reports employment with AstraZeneca at the time of the study work, and stock ownership with AstraZeneca. MP reports, outside the submitted work, personal fees from Rafael and RenovoRx; grants from Bavarian Nordic, Abbvie, Celldex, Pfizer, Novartis, Boston Biomedical, Tesaro, BMS, Genetech, ARMO Biosciences, Bayer, Calithera, Curegenix, Fibrogen, Gilead, GSK, Karyopharm, Regeneron, and Pharmacyclics; personal fees and nonfinancial support from Sirtex Medical, Caris Life Sciences, Ipsen/Merrimack, and Perthera, Inc; and grants, personal fees, and nonfinancial support from Merck, Astra Zeneca/Medimmune, Halozyme, and Celgene.

## DISCLOSURES

The study was conducted by Optum, by contract with AstraZeneca. At the time of the study, YD and GA were employees of US Medical Affairs, AstraZeneca; GA is currently employed by Regeneron. CB‐P and LL are employees of Health Economics and Outcomes Research, Optum. During the study, MP was employed by the Lombardi Cancer Center, Georgetown University, and is currently employed by the NCR Kimmel Cancer Center, Sibley Memorial Hospital and Johns Hopkins University School of Medicine.

## AUTHOR CONTRIBUTIONS

YD, LSL: Conceptualization and design; analysis and interpretation; manuscript revision. CB‐P: Acquisition, analysis, and interpretation; and manuscript revision. GA, MP: Conceptualization and design; interpretation; and manuscript revision. All authors approved the final version of the manuscript.

## Supporting information

Table S1‐S5

## Data Availability

The data contained in the claims database contain proprietary elements owned by Optum and, therefore, cannot be broadly disclosed or made publicly available at this time. The disclosure of these data to third‐party clients assumes certain data security and privacy protocols are in place and that the third party client has executed our standard license agreement which includes restrictive covenants governing the use of the data.
