# Supplementary material for: Treatment patterns and outcomes in pancreatic cancer: Retrospective claims analysis
Source: Cancer Med. 2020 Mar 25;9(10):3463–76. doi: 10.1002/cam4.3011 (PMC7221424; doi:10.1002/cam4.3011)
Supplement: Supplementary file 1 — Table S1‐S5 [file CAM4-9-3463-s001.docx]

**APPENDIX**

Table A1: Codes for Pancreatic Cancer Diagnoses

| **ICD-9-Dx Code** | **ICD-10-Dx Code** | **Description** |
| --- | --- | --- |
| 157.0 | C250 | Malignant neoplasm of head of pancreas |
| 157.1 | C251 | Malignant neoplasm of body of pancreas |
| 157.2 | C252 | Malignant neoplasm of tail of pancreas |
| 157.3 | C253 | Malignant neoplasm of pancreatic duct |
|  | C257 | Malignant neoplasm of other parts of pancreas |
|  | C258 | Malignant neoplasm of overlapping sites of pancreas |
|  | C259 | Malignant neoplasm of pancreas, unspecified |
| 157.8 |  | Malignant neoplasm of other specified sites of pancreas |
| 157.9 |  | Malignant neoplasm of pancreas, part unspecified |

Table A2: Metastatic Diagnosis Codes

| **ICD-9-Dx Code** | **ICD-10-Dx Code** | **Description** |
| --- | --- | --- |
| 196 | C770 | Secondary and unspecified malignant neoplasm of lymph nodes of head, face, and neck |
| 196.1 | C771 | Secondary and unspecified malignant neoplasm of intrathoracic lymph nodes |
| 196.2 | C772 | Secondary and unspecified malignant neoplasm of intra-abdominal lymph nodes |
| 196.3 | C773 | Secondary and unspecified malignant neoplasm of lymph nodes of axilla and upper limb |
| 196.5 | C774 | Secondary and unspecified malignant neoplasm of lymph nodes of inguinal region and lower limb |
| 196.6 | C775 | Secondary and unspecified malignant neoplasm of intrapelvic lymph nodes |
| 196.8 | C778 | Secondary and unspecified malignant neoplasm of lymph nodes of multiple sites |
| 196.9 | C779 | Secondary and unspecified malignant neoplasm of lymph nodes, site unspecified |
| 197 |  | Secondary malignant neoplasm of lung |
|  | C7800 (C7801, C7802) | Secondary malignant neoplasm of unspecified (right, left) lung |
| 197.1 | C781 | Secondary malignant neoplasm of mediastinum |
| 197.2 | C782 | Secondary malignant neoplasm of pleura |
|  | C7830 | Secondary malignant neoplasm of unspecified respiratory organ |
| 197.3 | C7839 | Secondary malignant neoplasm of other respiratory organs |
| 197.4 | C784 | Secondary malignant neoplasm of small intestine including duodenum |
| 197.5 | C785 | Secondary malignant neoplasm of large intestine and rectum |
| 197.6 | C786 | Secondary malignant neoplasm of retroperitoneum and peritoneum |
| 197.7 |  | Secondary malignant neoplasm of liver |
|  | C787 | Secondary malignant neoplasm of liver and intrahepatic bile duct |
|  | C7880 | Secondary malignant neoplasm of unspecified digestive organ |
| 197.8 |  | Secondary malignant neoplasm of other digestive organs and spleen |
|  | C7889 | Secondary malignant neoplasm of other digestive organs |
| 198 |  | Secondary malignant neoplasm of kidney |
|  | C7900 (C7901, C7902) | Secondary malignant neoplasm of unspecified (right, left) kidney and renal pelvis |
|  | C7910 | Secondary malignant neoplasm of unspecified urinary organs |
|  | C7911 | Secondary malignant neoplasm of bladder |
| 198.1 | C7919 | Secondary malignant neoplasm of other urinary organs |
| 198.2 | C792 | Secondary malignant neoplasm of skin |
| 198.3 |  | Secondary malignant neoplasm of brain and spinal cord |
|  | C7931 | Secondary malignant neoplasm of brain |
|  | C7932 | Secondary malignant neoplasm of cerebral meninges |
|  | C7940 | Secondary malignant neoplasm of unspecified part of nervous system |
| 198.4 | C7949 | Secondary malignant neoplasm of other parts of nervous system |
| 198.5 |  | Secondary malignant neoplasm of bone and bone marrow |
|  | C7951 | Secondary malignant neoplasm of bone |
|  | C7952 | Secondary malignant neoplasm of bone marrow |
| 198.6 |  | Secondary malignant neoplasm of ovary |
|  | C7960 (C7961, C7962) | Secondary malignant neoplasm of unspecified (right, left) ovary |
| 198.7 |  | Secondary malignant neoplasm of adrenal gland |
|  | C7970 (C7971, C7972) | Secondary malignant neoplasm of unspecified (right, left) adrenal gland |
| 198.82 | C7982 | Secondary malignant neoplasm of genital organs |
| 198.89 | C7989 | Secondary malignant neoplasm of other specified sites |
|  | C799 | Secondary malignant neoplasm of unspecified site |
| 199 | C800 | Disseminated malignant neoplasm |

Table A3: Systemic Therapies for Cancer: Procedure Codes

| **Pharmacological Category** | **Medication (generic name)** | **HCPCS** | **HCPCS description** |
| --- | --- | --- | --- |
| Alkylating agents, nitrosoureas | Carmustine | J9050 | Injection, carmustine, 100 mg |
| Biological response modifiers, chemotherapeutic | Interferon gamma-1b | J9216 | Injection, interferon, gamma 1-b, 3 million units |
| Anthracycline antibiotics | Epirubicin hydrochloride | J9178 | Injection, epirubicin HCl, 2 mg |
|  |  | J9180 | Epirubicin hydrochloride, 50 mg |
| Mitotic inhibitors, podophyllotoxin derivatives | Etoposide | J9181 | Injection, etoposide, 10 mg |
|  |  | J9182 | Etoposide 100 mg |
| Antimetabolites | Fludarabine phosphate | J9185 | Injection, fludarabine phosphate, 50 mg |
|  | Fluorouracil | J9190 | Injection, fluorouracil, 500 mg |
|  | Floxuridine | J9200 | Injection, floxuridine, 500 mg |
|  | Gemcitabine hydrochloride | J9201 | Injection, gemcitabine HCl, 200 mg |
| Topoisomerase inhibitors | Irinotecan | J9206 | Injection, irinotecan, 20 mg |
| Alkylating agents, nitrogen mustards | Ifosfamide | J9208 | Injection, ifosfamide, 1 g |
| Anthracycline antibiotics | Idarubicin hydrochloride | J9211 | Injection, idarubicin HCl, 5 mg |
| Biological response modifiers, chemotherapeutic | Interferon alfacon-1 | J9212 | Injection, interferon alfacon-1, recombinant, 1 mcg |
|  | Interferon alfa-2a [recombinant] | J9213 | Injection, interferon, alfa-2a, recombinant, 3 million units |
|  | Interferon alfa-2b [recombinant] | J9214 | Injection, interferon, alfa-2b, recombinant, 1 million units |
|  | Interferon alfa-n3 [human leukocyte-derived] | J9215 | Injection, interferon, alfa-N3, (human leukocyte derived), 250,000 IU |
| Alkylating agents, nitrogen mustards | Mechlorethamine hydrochloride | J9230 | Injection, mechlorethamine HCl, (nitrogen mustard), 10 mg |
|  | Melphalan | J9245 | Injection, melphalan HCl, 50 mg |
| Antimetabolites | Methotrexate sodium | J9250 | Methotrexate sodium, 5 mg |
|  |  | J9260 | Methotrexate sodium, 50 mg |
| Alkylating agents, miscellaneous | Oxaliplatin | J9263 | Injection, oxaliplatin, 0.5 mg |
| Mitotic inhibitors, taxanes | Paclitaxel, protein-bound particles | J9264 | Injection, paclitaxel protein-bound particles, 1 mg |
|  | Paclitaxel | J9265 | Injection, paclitaxel, 30 mg |
| Enzymes, chemotherapeutic | Pegaspargase | J9266 | Injection, pegaspargase, per single dose vial |
| Antineoplastic antibiotics, miscellaneous | Pentostatin, injectable | J9268 | Injection, pentostatin, 10 mg |
|  | Plicamycin | J9270 | Injection, plicamycin, 2.5 mg |
|  | Mitomycin | J9280 | Injection, mitomycin, 5 mg |
|  |  | J9290 | Mitomycin, 20 mg |
|  |  | J9291 | Mitomycin, 40 mg |
|  | Mitoxantrone hydrochloride | J9293 | Injection, mitoxantrone HCl, per 5 mg |
| Monoclonal antibodies, chemotherapeutic | Gemtuzumab ozogamicin | J9300 | Injection, gemtuzumab ozogamicin, 5 mg |
| Antimetabolites | Pemetrexed | J9305 | Injection, pemetrexed, 10 mg |
| Monoclonal antibodies, chemotherapeutic | Rituximab | J9310 | Injection, rituximab, 100 mg |
| Alkylating agents, nitrosoureas | Streptozocin | J9320 | Injection, streptozocin, 1 g |
| Alkylating agents, miscellaneous | Thiotepa | J9340 | Injection, thiotepa, 15 mg |
| Topoisomerase inhibitors | Topotecan | J9350 | Injection, topotecan, 4 mg |
| Monoclonal antibodies, chemotherapeutic | Trastuzumab | J9355 | Injection, trastuzumab, 10 mg |
| Anthracycline antibiotics | Valrubicin | J9357 | Injection, valrubicin, intravesical, 200 mg |
| Mitotic inhibitors, vinca alkaloids | Vinblastine sulfate | J9360 | Injection, vinblastine sulfate, 1 mg |
|  |  | J9370 | Vincristine sulfate, 1 mg |
|  |  | J9375 | Vincristine sulfate, 2 mg |
|  |  | J9380 | Vincristine sulfate, 5 mg |
|  | Vinorelbine tartrate | J9390 | Injection, vinorelbine tartrate, 10 mg |
| Antineoplastics, miscellaneous | Porfimer sodium | J9600 | Injection, porfimer sodium, 75 mg |
| Mitotic inhibitors, podophyllotoxin derivatives | Teniposide | Q2017 | Injection, teniposide, 50 mg |
| Monoclonal antibodies, chemotherapeutic | Alemtuzumab (campath) | S0087 | Injection alemtuzumab |
| Kinase inhibitors, chemotherapeutic | Imatinib mesylate | S0088 | Imatinib, 100 mg |
| Antimetabolites | Mercaptopurine | S0108 | Mercaptopurine, oral, 50 mg |
| Proteasome inhibitors | Bortezomib | S0115 | Bortezomib, 3.5 mg |
| Monoclonal antibodies, chemotherapeutic | Bevacizumab | S0116 | Bevacizumab 100 mg |
| Biological response modifiers, chemotherapeutic | Peginterferon alfa-2a | S0145 | Injection, pegylated interferon alfa-2a, 180 mcg per ml |
|  | Peginterferon alfa-2b | S0146 | Injection, pegylated interferon alfa-2b, 10 mcg per 0.5 ml |
| Antimetabolites | Azacitidine | S0168 | Injection, azacitidine, 100 mg |
| Alkylating agents, nitrogen mustards | Chlorambucil | S0172 | Chlorambucil, oral, 2 mg |
| Antineoplastics, miscellaneous | Hydroxyurea | S0176 | Hydroxyurea, oral, 500 mg |
|  | Levamisole hydrochloride | S0177 | Levamisole HCl, oral, 50 mg |
| Alkylating agents, nitrosoureas | Lomustine | S0178 | Lomustine, oral, 10 mg |
| Antineoplastics, miscellaneous | Procarbazine hydrochloride | S0182 | Procarbazine HCl, oral, 50 mg |
| Monoclonal antibodies, chemotherapeutic | Ibritumomab tiuxetan & yttrium-90 [y-90] & indium-111 [I-111] | S8003 | Supply of therapeutic radioimmunopharmaceutical, |
| Alkylating agents, miscellaneous | Busulfan | J8510 | Busulfan, oral, 2 mg |
| Antimetabolites | Capecitabine | J8520 | Capecitabine, oral, 150 mg |
|  | Capecitabine | J8521 | Capecitabine, oral, 500 mg |
| Alkylating agents, nitrogen mustards | Cyclophosphamide | J8530 | Cyclophosphamide, oral, 25 mg |
| Mitotic inhibitors, podophyllotoxin derivatives | Etoposide | J8560 | Etoposide, oral, 50 mg |
| Kinase inhibitors, chemotherapeutic | Gefitinib | J8565 | Gefitinib, oral, 250 mg |
| Alkylating agents, nitrogen mustards | Melphalan | J8600 | Melphalan, oral, 2 mg |
| Antimetabolites | Methotrexate sodium | J8610 | Methotrexate, oral, 2.5 mg |
| Antineoplastics, miscellaneous | Temozolomide | J8700 | Temozolomide, oral, 5 mg |
| Anthracycline antibiotics | Doxorubicin hydrochloride | J9000 | Injection, doxorubicin HCl, 10 mg |
|  | Doxorubicin liposomal | J9001 | Injection, doxorubicin HCl, all lipid formulations, 10 mg |
| Monoclonal antibodies, chemotherapeutic | Alemtuzumab (campath) | J9010 | Injection, alemtuzumab, 10 mg |
| Biological response modifiers, chemotherapeutic | Aldesleukin | J9015 | Injection, aldesleukin, per single use vial |
| Antineoplastics, miscellaneous | Arsenic trioxide | J9017 | Injection, arsenic trioxide, 1 mg |
| Enzymes, chemotherapeutic | Asparaginase | J9020 | Injection, asparaginase, not otherwise specified, 10,000 units |
| Antimetabolites | Azacitidine | J9025 | Injection, azacitidine, 1 mg |
|  | Clofarabine | J9027 | Injection, clofarabine, 1 mg |
| Vaccines, chemotherapeutic | Bacillus of calmette & guerin (BCG) vaccine, used as antineoplastic | J9031 | BCG (intravesical) per instillation |
| Monoclonal antibodies, chemotherapeutic | Bevacizumab | J9035 | Injection, bevacizumab, 10 mg |
| Antineoplastic antibiotics, miscellaneous | Bleomycin sulfate | J9040 | Injection, bleomycin sulfate, 15 units |
| Proteasome inhibitors | Bortezomib | J9041 | Injection, bortezomib, 0.1 mg |
| Alkylating agents, miscellaneous | Carboplatin | J9045 | Injection, carboplatin, 50 mg |
| Monoclonal antibodies, chemotherapeutic | Cetuximab | J9055 | Injection, cetuximab, 10 mg |
| Alkylating agents, miscellaneous | Cisplatin | J9060 | Injection, cisplatin, powder or solution, 10 mg |
|  |  | J9062 | Cisplatin, 50 mg |
| Antimetabolites | Cladribine | J9065 | Injection, cladribine, per 1 mg |
| Alkylating agents, nitrogen mustards | Cyclophosphamide | J9070 | Cyclophosphamide, 100 mg |
|  |  | J9080 | Cyclophosphamide, 200 mg |
|  |  | J9090 | Cyclophosphamide, 500 mg |
|  |  | J9091 | Cyclophosphamide, 1 g |
|  |  | J9092 | Cyclophosphamide, 2 g |
|  |  | J9093 | Cyclophosphamide, lyophilized, 100 mg |
|  |  | J9094 | Cyclophosphamide, lyophilized, 200 mg |
|  |  | J9095 | Cyclophosphamide, lyophilized, 500 mg |
|  |  | J9096 | Cyclophosphamide, lyophilized, 1 g |
|  |  | J9097 | Cyclophosphamide, lyophilized, 2 g |
| Antimetabolites | Cytarabine liposomal | J9098 | Injection, cytarabine liposome, 10 mg |
|  | Cytarabine | J9100 | Injection, cytarabine, 100 mg |
|  |  | J9110 | Injection, cytarabine, 500 mg |
| Antineoplastic antibiotics, miscellaneous | Dactinomycin | J9120 | Injection, dactinomycin, 0.5 mg |
| Alkylating agents, miscellaneous | Dacarbazine | J9130 | Dacarbazine, 100 mg |
|  |  | J9140 | Dacarbazine, 200 mg |
| Anthracycline antibiotics | Daunorubicin hydrochloride | J9150 | Injection, daunorubicin, 10 mg |
|  | Daunorubicin citrate, liposomal | J9151 | Injection, daunorubicin citrate, liposomal formulation, 10 mg |
| Biological response modifiers, chemotherapeutic | Denileukin diftitox | J9160 | Injection, denileukin diftitox, 300 mcg |
| Mitotic inhibitors, taxanes | Docetaxel | J9170 | Injection, docetaxel, 20 mg |
| Monoclonal antibodies, chemotherapeutic | Tositumomab & iodine I-131 i-tositumomab | G3001 | Administration and supply of tositumomab, 450 mg |
| Folic or folinic acid products | Leucovorin calcium | J0640 | Injection, leucovorin calcium, per 50 mg |
| Monoclonal antibodies, chemotherapeutic | Ibritumomab tiuxetan & yttrium-90 [y-90] & indium-111 [I-111] | A9543 | Yttrium Y-90 ibritumomab tiuxetan, therapeutic, per treatment dose, up to 40 millicuries |
|  | Tositumomab & iodine I-131 i-tositumomab | A9545 | Iodine I-131 tositumomab, therapeutic, per treatment dose |
| Antineoplastic radiopharmaceuticals | Sodium phosphate P-32 | A9563 | Sodium phosphate P-32, therapeutic, per millicurie |
|  | Chromic phosphate P-32 | A9564 | Chromic phosphate P-32 suspension, therapeutic, per millicurie |
|  | Strontium-89 chloride | A9600 | Strontium Sr-89 chloride, therapeutic, per millicurie |
|  | Samarium SM 153 lexidronam | A9605 | Samarium Sm-153 lexidronamm, therapeutic, per 50 millicuries |
| Monoclonal antibodies, chemotherapeutic | Alemtuzumab (campath) | C9110 | Almetuzumab |
|  | Ibritumomab tiuxetan & yttrium-90 [y-90] & indium-111 [I-111] | C9117 | Injection, yttrium 90 ibritumomab tiuxetan, per 50 millicuries |
|  | Ibritumomab tiuxetan & yttrium-90 [y-90] & indium-111 [I-111] | C9118 | Injection, indium 111 ibritumomab tiuxetan, per 50 millicuries |
| Mitotic inhibitors, taxanes | Paclitaxel, protein-bound particles | C9127 | Injection, paclitaxel protein-bound particles |
| Antimetabolites | Clofarabine | C9129 | Injection, clofarabine, per 1 mg |
| Alkylating agents, miscellaneous | Oxaliplatin | C9205 | Oxaliplatin injection, oxaliplatin, per 5 mg |
| Proteasome inhibitors | Bortezomib | C9207 | Injection, iv, bortezomib, per 3.5 mg |
| Antimetabolites | Pemetrexed | C9213 | Injection, pemetrexed, per 10 mg |
| Monoclonal antibodies, chemotherapeutic | Bevacizumab | C9214 | Injection, bevacizumab, per 10 mg |
|  | Cetuximab | C9215 | Injection, cetuximab, per 10 mg |
| Antimetabolites | Azacitidine | C9218 | Injection, azacitidine, per 1 mg |
| Antineoplastic radiopharmaceuticals | Strontium-89 chloride | C9401 | Supply of therapeutic radiopharm-aceutical strontium-89 c |
|  | Sodium iodide I-131 | C9402 | Supply of radiopharmaceutical therapeutic imaging |
| Mitotic inhibitors, podophyllotoxin derivatives | Etoposide | C9414 | Etoposide; oral, 50 mg, brand name |
| Anthracycline antibiotics | Doxorubicin hydrochloride | C9415 | Doxorubicin HCl, 10 mg, brand name |
| Vaccines, chemotherapeutic | Bacillus of calmette & guerin (BCG) vaccine, used as antineoplastic | C9416 | BCG (intravesical) per instillation, brand name |
| Antineoplastic antibiotics, miscellaneous | Bleomycin sulfate | C9417 | Bleomycin sulfate, 15 units, brand name |
| Alkylating agents, miscellaneous | Cisplatin | C9418 | Cisplatin, powder or solution, per 10 mg, brand |
| Antimetabolites | Cladribine | C9419 | Injection, cladribine, per 1 mg, brand |
| Alkylating agents, nitrogen mustards | Cyclophosphamide | C9420 | Cyclophosphamide, 100 mg, brand |
|  |  | C9421 | Cyclophosphamide, lyophilized, 100 mg, brand name |
| Antimetabolites | Cytarabine | C9422 | Cytarabine, 100 mg, brand name |
| Alkylating agents, miscellaneous | Dacarbazine | C9423 | Dacarbazine, 100 mg, brand name |
| Anthracycline antibiotics | Daunorubicin hydrochloride | C9424 | Daunorubicin hcl, 10 mg, brand name |
| Mitotic inhibitors, podophyllotoxin derivatives | Etoposide | C9425 | Etoposide, 10 mg, brand name |
| Antimetabolites | Floxuridine | C9426 | Floxuridine, 500 mg, brand name |
| Alkylating agents, nitrogen mustards | Ifosfamide | C9427 | Ifosfamide, 1 gm, brand name |
| Anthracycline antibiotics | Idarubicin hydrochloride | C9429 | Idarubicin hydrochloride, 5 mg, brand name |
| Mitotic inhibitors, taxanes | Paclitaxel | C9431 | Paclitaxel, 30 mg, brand name |
| Antineoplastic antibiotics, miscellaneous | Mitomycin | C9432 | Mitomycin, 5 mg, brand name |
|  | Thiotepa | C9433 | Thiotepa, 15 mg, brand name |
| Alkylating agents, nitrosoureas | Carmustine | C9437 | Carmustine, brand name, 100 mg |
| Mitotic inhibitors, vinca alkaloids | Vinorelbine tartrate | C9440 | Vinorelbine tartrate, brand name, per 10 mg |
| Antineoplastic radiopharmaceuticals | Sodium iodide I-131 | A9517 | Iodine I-131 sodium iodide capsule(s), therapeutic, per millicurie |
| Monoclonal antibodies, chemotherapeutic | Ibritumomab tiuxetan & yttrium-90 [y-90] & indium-111 [I-111] | A9522 | Supply-radiopharm dx imaging agt, indium-111 ibritumomabtiuxetan |
|  | Ibritumomab tiuxetan & yttrium-90 [y-90] & indium-111 [I-111] | A9523 | Supply-radiopharm therapeutic imaging agt, yttrium-111 ibritumomabtiuxetan |
| Antineoplastic radiopharmaceuticals | Sodium iodide I-131 | A9530 | Iodine I-131 sodium iodide solution, therapeutic, per millicurie |
| Monoclonal antibodies, chemotherapeutic | Tositumomab & iodine I-131 i-tositumomab | A9534 | Supply of radiopharmaceutical therapeutic imaging I-131 tositumomab |
| Antineoplastic radiopharmaceuticals | Sodium iodide I-131 | C1064 | Supply of radiopharmaceutical therapeutic imaging |
| Monoclonal antibodies, chemotherapeutic | Tositumomab & iodine I-131 i-tositumomab | C1081 | Supply of radiopharmaceutical therapeutic imaging |
|  | Ibritumomab tiuxetan & yttrium-90 [y-90] & indium-111 [I-111] | C1082 | Supply of radiopharmaceutical diag imaging agt, In-111 ibritumomab tiuxetan |
|  | Ibritumomab tiuxetan & yttrium-90 [y-90] & indium-111 [I-111] | C1083 | Supply of radiopharmaceut therap imaging agt, Yttrium 90 ibritumomab |
| Antimetabolites | Cytarabine liposomal | C1166 | Injection, cytarabine liposome, per 10 mg |
| Anthracycline antibiotics | Epirubicin hydrochloride | C1167 | Injection, epirubicin HCl, 2 mg |
| Alkylating agents, miscellaneous | Busulfan | C1178 | Injection busulfan per 6 mg |
| Antineoplastic radiopharmaceuticals | Sodium iodide I-131 | A9527 | Iodine I-125, sodium iodide solution, therapeutic, per millicurie |
| Monoclonal antibodies, chemotherapeutic | Panitumumab | C9235 | Inj panitumumab 10 mg |
| Alkylating agents, miscellaneous | Busulfan | J0594 | Injection, busulfan, 1 mg |
| Antimetabolites | Decitabine | J0894 | Injection, decitabine, 1 mg |
|  | Nelarabine | J9261 | Injection, nelarabine, 50 mg |
|  | Decitabine | C9231 | Injection decitabine per 1 mg |
| Monoclonal antibodies, chemotherapeutic | Panitumumab | J9303 | Injection, panitumumab, 10 mg |
| MTOR inhibitors | Temsirolimus | C9239 | Injection temsirolimus 1 mg |
| Mitotic inhibitors, epothilones | Ixabepilone | C9240 | Injection ixabepilone 1 mg |
| Topoisomerase inhibitors | Topotecan | J8705 | Topotecan, oral, 0.25 mg |
| Alkylating agents, nitrogen mustards | Bendamustine hydrochloride | J9033 | Injection, bendamustine HCl, 1 mg |
| Mitotic inhibitors, epothilones | Ixabepilone | J9207 | Injection, ixabepilone, 1 mg |
| MTOR inhibitors | Temsirolimus | J9330 | Injection, temsirolimus, 1 mg |
| Alkylating agents, nitrogen mustards | Bendamustine hydrochloride | C9243 | Injection bendamustine hcl 1 mg |
| Antineoplastics, miscellaneous | Temozolomide | C9253 | Injection, temozolomide, 1mg |
| Mitotic inhibitors, taxanes | Docetaxel | J9171 | Injection, docetaxel, 1 mg |
| Antineoplastics, miscellaneous | Temozolomide | J9328 | Injection, temozolomide, 1 mg |
| Antimetabolites | Fludarabine phosphate | C9262 | Fludarabine phosphate, oral, 1 mg |
|  | Fludarabine phosphate | J8562 | Fludarabine phosphate, oral, 10 mg |
|  | Fludarabine phosphate | Q2025 | Fludarabine phosphate, oral, 1 mg |
|  | Pralatrexate | C9259 | Injection, pralatrexate, 1 mg |
|  | Pralatrexate | J9307 | Injection, pralatrexate, 1 mg |
| Topoisomerase inhibitors | Topotecan | J9351 | Injection, topotecan, 0.1 mg |
| Mitotic inhibitors, taxanes | Cabazitaxel | C9276 | Injection, cabazitaxel, 1 mg |
| Biological response modifiers, chemotherapeutic | Peginterferon alfa-2b | S0148 | Injection, pegylated interferon alfa-2B, 10 mcg |
| Histone deacetylase inhibitors | Romidepsin | C9265 | Injection, romidepsin, 1 mg |
|  | Romidepsin | J9315 | Injection, romidepsin, 1 mg |
| Monoclonal antibodies, chemotherapeutic | Ofatumumab | C9260 | Injection, ofatumumab, 10 mg |
|  | Ofatumumab | J9302 | Injection, ofatumumab, 10 mg |
| Vaccines, chemotherapeutic | Sipuleucel-T | C9273 | Sipuleucel-T, minimum of 50 million autologous CD54+ cells activated with PAP-GM-CSF, including leukapheresis and all other preparatory procedures, per infusion |
| Anthracycline antibiotics | Doxorubicin liposomal | J9002 | Injection, doxorubicin hydrochloride, liposomal, Doxil, 10 mg |
| Enzymes, chemotherapeutic | Asparaginase | J9019 | Injection, asparaginase (Erwinaze), 1,000 IU |
| Monoclonal antibodies, chemotherapeutic | Brentuximab vedotin | J9042 | Injection, brentuximab vedotin, 1 mg |
| Anthracycline antibiotics | Doxorubicin liposomal | Q2048 | Injection, doxorubicin hydrochloride, liposomal, DOXIL, 10 mg |
|  |  | Q2049 | Injection, doxorubicin hydrochloride, liposomal, imported Lipodox, 10 mg |
| Mitotic inhibitors, halichondrin b analogs | Eribulin mesylate | C9280 | Injection, eribulin mesylate, 1 mg |
| Monoclonal antibodies, chemotherapeutic | Ipilimumab | C9284 | Injection, ipilimumab, 1 mg |
|  | Brentuximab vedotin | C9287 | Injection, brentuximab vedotin, 1 mg |
| Mitotic inhibitors, taxanes | Cabazitaxel | J9043 | Injection, cabazitaxel, 1 mg |
| Mitotic inhibitors, halichondrin b analogs | Eribulin mesylate | J9179 | Injection, eribulin mesylate, 0.1 mg |
| Monoclonal antibodies, chemotherapeutic | Ipilimumab | J9228 | Injection, ipilimumab, 1 mg |
| Vaccines, chemotherapeutic | Sipuleucel-T | Q2043 | Sipuleucel-T, minimum of 50 million autologous cd54+ cells activated with PAP-GM-CSF, including leukapheresis and all other preparatory procedures, per infusion |
| Enzymes, chemotherapeutic | Asparaginase | C9289 | Injection, asparaginase Erwinia chrysanthemi, 1,000 IU |
| Monoclonal antibodies, chemotherapeutic | Pertuzumab | C9292 | Injection, pertuzumab, 10 mg |
| Proteasome inhibitors | Carfilzomib | C9295 | Injection, carfilzomib, 1 mg |
| Antineoplastics, miscellaneous | Ziv-aflibercept, intravenous | C9296 | Injection, ziv-aflibercept, 1 mg |
| Anthracycline antibiotics | Doxorubicin liposomal | Q2050 | Injection, doxorubicin hydrochloride, liposomal, NOS, 10 mg |
| Mitotic inhibitors, vinca alkaloids | Vincristine sulfate | J9371 | Injection, vincristine sulfate liposome, 1 mg |
| Proteasome inhibitors | Carfilzomib | J9047 | Injection, carfilzomib, 1 mg |
| Antineoplastics, miscellaneous | Ziv-aflibercept, intravenous | J9400 | Injection, ziv-aflibercept, 1 mg |
|  | Omacetaxine mepesuccinate | C9297 | Injection, omacetaxine mepesuccinate, 0.01 mg |
|  | Omacetaxine mepesuccinate | J9262 | Injection, omacetaxine mepesuccinate, 0.01 mg |
| Monoclonal antibodies, chemotherapeutic | Pertuzumab | J9306 | Injection, pertuzumab, 1 mg |
|  | Ado-trastuzumab emtansine | C9131 | Injection, ado-trastuzumab emtansine, 1 mg |
|  | Ado-trastuzumab emtansine | J9354 | Injection, ado-trastuzumab emtansine, 1 mg |
|  | Obinutuzumab | C9021 | Injection, obinutuzumab, 10 mg |
| Antineoplastic radiopharmaceuticals | Radium Ra-223 dichloride | A9606 | Radium RA-223 dichloride, therapeutic, per microcurie |
| Monoclonal antibodies, chemotherapeutic | Ramucirumab | C9025 | Injection, ramucirumab, 5 mg |
|  | Pembrolizumab | C9027 | Injection, pembrolizumab, 1 mg |
| Histone deacetylase inhibitors | Belinostat | C9442 | Injection, belinostat, 10 mg |
| Mitotic inhibitors, taxanes | Paclitaxel | J9267 | Injection, paclitaxel, 1 mg |
| Monoclonal antibodies, chemotherapeutic | Obinutuzumab | J9301 | Injection, obinutuzumab, 10 mg |
|  | Blinatumomab | C9449 | Injection, blinatumomab, 1 mcg |
|  | Nivolumab | C9453 | Injection, nivolumab, 1 mg |
| Histone deacetylase inhibitors | Belinostat | J9032 | Injection, belinostat, 10 mg |
| Monoclonal antibodies, chemotherapeutic | Blinatumomab | J9039 | Injection, blinatumomab, 1 mcg |
|  | Pembrolizumab | J9271 | Injection, pembrolizumab, 1 mg |
|  | Nivolumab | J9299 | Injection, nivolumab, 1 mg |
|  | Ramucirumab | J9308 | Injection, ramucirumab, 5 mg |
| Oncolytic virotherapies | Talimogene laherparepvec | C9472 | Injection, talimogene laherparepvec, 1 million plaque forming units (PFU) |
| Topoisomerase inhibitors | Irinotecan | C9474 | Injection, irinotecan liposome, 1 mg |
| Monoclonal antibodies, chemotherapeutic | Necitumumab | C9475 | Injection, necitumumab, 1 mg |
|  | Daratumumab | C9476 | Injection, daratumumab, 10 mg |
|  | Elotuzumab | C9477 | Injection, elotuzumab, 1 mg |
| Alkylating agents, miscellaneous | Trabectedin | C9480 | Injection, trabectedin, 0.1 mg |
| Monoclonal antibodies, chemotherapeutic | Atezolizumab | C9483 | Injection, atezolizumab, 10 mg |
| Alkylating agents, nitrogen mustards | Bendamustine HCl | J9034 | Injection, bendamustine HCl, 1 mg |
| Monoclonal antibodies, chemotherapeutic | Daratumumab | J9145 | Injection, daratumumab, 10 mg |
|  | Elotuzumab | J9176 | Injection, elotuzumab, 1 mg |
| Topoisomerase inhibitors | Irinotecan | J9205 | Injection, irinotecan liposome, 1 mg |
| Monoclonal antibodies, chemotherapeutic | Necitumumab | J9295 | Injection, necitumumab, 1 mg |
| Oncolytic virotherapies | Talimogene laherparepvec | J9325 | Injection, talimogene laherparepvec, per 1 million plaque forming units |
| Alkylating agents, miscellaneous | Trabectedin | J9352 | Injection, trabectedin, 0.1 mg |

*Note*. HCPCS=Healthcare Common Procedure Coding System

**Table A4.** Regimens Included in “Other” Category, Used with Frequency >1% (n=1,613 Patients)

| **Regimen** | **Frequency (n)** | **Proportion (%)** |
| --- | --- | --- |
| Capecitabine | 156 | 9.7 |
| Cisplatin, gemcitabine | 153 | 9.5 |
| Fluorouracil | 132 | 8.2 |
| Erlotinib, gemcitabine | 126 | 7.8 |
| Gemcitabine, oxaliplatin | 73 | 4.5 |
| Carboplatin, etoposide | 64 | 4.0 |
| Capecitabine, gemcitabine | 63 | 3.9 |
| Everolimus | 52 | 3.2 |
| Capecitabine, docetaxel, gemcitabine | 46 | 2.9 |
| Carboplatin, paclitaxel | 37 | 2.3 |
| Sunitinib | 34 | 2.1 |
| Fluorouracil, oxaliplatin | 33 | 2.1 |
| Cyclophosphamide, doxorubicin, rituximab, vincristine | 30 | 1.9 |
| Carboplatin, gemcitabine | 26 | 1.6 |
| Capecitabine, temozolomide | 22 | 1.4 |
| Cisplatin, etoposide | 21 | 1.3 |
| Docetaxel, gemcitabine | 21 | 1.3 |

**Table A5.** Mean Costs per Patient per Month (PPPM) by First-Line Systemic Therapy Regimen

|  | **Total Sample (N=12,978)** | **Gem-*nab*-P (n=1,280)** | **FOLFIRINOX (n=1,234)** | **Gem (n=1,322)** | **FOLFOX (n=161)** | **Others (n=1,613)** | **No First Line  (n=7,368)** |
| --- | --- | --- | --- | --- | --- | --- | --- |
| **Full Follow-up Period Costs, mean PPPM, $US** | | | | | | | |
| Cost Category |  |  |  |  |  |  |  |
| Office visits | $1,166 | $2,916 | $3,033 | $1,457 | $2,035 | $2,210 | $249 |
| Outpatient visits | $3,004 | $5,361 | $8,746 | $3,146 | $5,082 | $5,051 | $1,113 |
| ER visits | $262 | $267 | $209 | $226 | $204 | $203 | $291 |
| Inpatient costs | $8,238 | $5,175 | $5,458 | $5,810 | $8,038 | $6,451 | $10,067 |
| Other medical costs | $466 | $531 | $901 | $307 | $595 | $763 | $342 |
| Pharmacy costs | $578 | $627 | $988 | $535 | $845 | $1,594 | $281 |
| Total healthcare costs | $13,713 | $14,876 | $19,335 | $11,482 | $16,801 | $16,271 | $12,342 |
| **6-Month Follow-up Period Costs, mean PPPM, $US** | | | | | | | |
| Cost Category |  |  |  |  |  |  |  |
| Office visits | $1,360 | $3,365 | $3,766 | $1,626 | $2,143 | $2,635 | $265 |
| Outpatient visits | $3,504 | $6,229 | $10,788 | $3,603 | $5,808 | $5,971 | $1,202 |
| ER visits | $264 | $274 | $203 | $226 | $210 | $206 | $294 |
| Inpatient costs | $8,646 | $5,218 | $5,342 | $6,126 | $7,964 | $6,652 | $10,698 |
| Other medical costs | $489 | $542 | $954 | $312 | $656 | $716 | $381 |
| Pharmacy costs | $576 | $610 | $998 | $520 | $781 | $1,604 | $279 |
| Total healthcare costs | $14,839 | $16,239 | $22,051 | $12,413 | $17,561 | $17,785 | $13,119 |

## *Note:* Gem-*nab*-P = gemcitabine plus *nab*-paclitaxel; FOLFIRINOX = folinic acid [leucovorin], fluorouracil, irinotecan, and oxaliplatin; FOLFOX = folinic acid (leucovorin), fluorouracil, oxaliplatin;; PPPM = per patient per month; $US = United States dollar.
